# FIH permits NAA10 to catalyze the oxygen-dependent lysyl-acetylation of HIF-1α

**DOI:** 10.1016/j.redox.2018.09.002

**Published:** 2018-09-07

**Authors:** Jengmin Kang, Yang-Sook Chun, June Huh, Jong-Wan Park

**Affiliations:** a Department of Biomedical Science, BK21-plus education program, Seoul National University College of Medicine, Daehak-ro, Jongno-gu, Seoul 03080, Republic of Korea; b Department of Pharmacology, Seoul National University College of Medicine, Daehak-ro, Jongno-gu, Seoul 03080, Republic of Korea; c Cancer Research Institute and Ischemic/Hypoxic Disease Institute, Seoul National University College of Medicine, Daehak-ro, Jongno-gu, Seoul 03080, Republic of Korea; d Department of Chemical and Biological Engineering, Korea University, Anam-dong, Seongbuk-gu, Seoul 136-713, Republic of Korea

**Keywords:** FIH, NAA10, HIF-1α, Tryptophan hydroxylation, Lysine acetylation

## Abstract

The N-terminal acetyltransferase A (NatA) complex, which is composed of NAA10 and NAA15, catalyzes N-terminal acetylation of many proteins in a co-translational manner. Structurally, the catalytic subunit NAA10 was believed to have no activity toward an internal lysine residue because the gate of its catalytic pocket is too narrow. However, several studies have demonstrated that the monomeric NAA10 can acetylate the internal lysine residues of several substrates including hypoxia-inducible factor 1α (HIF-1α). How NAA10 acetylates lysine residues has been an unsolved question. We here found that human FIH (factor inhibiting HIF) hydroxylates human NAA10 at W38 oxygen-dependently and this permits NAA10 to express the lysyl-acetyltransferase activity. The hydroxylated W38 forms a new hydrogen-bond with A67 and widens the gate at the catalytic pocket, which allows the entrance of a lysine residue to the site. Since the FIH-dependent hydroxylation of NAA10 occurs oxygen-dependently, NAA10 acetylates HIF-1α under normoxia but does not under hypoxia. Consequently, the acetylation promotes the pVHL binding to HIF-1α, and in turn HIF-1α is destructed *via* the ubiquitin-proteasome system. This study provides a novel oxygen-sensing process that determines the substrate specificity of NAA10 depending on an ambient oxygen tension.

## 1 Introduction

Protein acetylation plays critical roles in many cellular processes including transcription, protein stability, chromatin remodeling, and metabolism [1]. Generally, protein acetylation is classified into two types: N-terminal α-acetylation at the co-translational step and internal ε-acetylation at the post-translational step [2], [3]. N-terminal acetylation is catalyzed by N-terminal acetyltransferases (NatA-G). Of them, NatA is composed of the catalytic subunit NAA10 (alternatively named arrest-defective 1, ARD1) and the auxiliary subunit NAA15. It selectively acetylates small amino acids, such as Ser, Ala, Thr, Gly, Val, and Cys, at the N-terminal ends of proteins [4]. The substrate specificity of this enzyme was further supported by a recent study suggesting that it hardly acetylates lysine residues because the entrance gate of its catalytic pocket is too narrow for a big lysine residue to enter the gate [5]. However, several reports have demonstrated that some of NAAs as a monomer have a moonlighting job to acetylate internal lysine residues of proteins. For instance, NAA50 acetylates itself at Lys34, Lys37 and Lys140 residues, thereby modulating its substrate specificity [6]. NAA10 has been also reported to acetylate lysine residues of several substrates, such as hypoxia-inducible factor-1α (HIF-1α), β-catenin, runt-related transcription factor 2, myosin light-chain kinase, methionine sulfoxide reductase A, and androgen receptor [7], [8], [9], [10], [11], [12]. Consequently, the NAA10-mediated lysine acetylation modulates stability or functionality of target proteins. Taken together, the mechanism by which monomeric NAA10 has the activity of lysine acetyltransferase remains an open question.

Although the NAA10-dependent lysine acetylation has been shown in diverse proteins, the argument of this NAA10 activity has been challenged by several reports showing that the HIF-1α acetylation was not reproducible *in vitro* [5], [13], [14], [15]. To understand such conflicting findings, Seo et al. searched for post-translational modifications of human NAA10 using MASS analysis, and found that NAA10 is lysyl-acetylated by itself and this autoacetylation is critical for the NAA10 function to activate β-catenine and AP-1 [16]. This report suggested the possibility that the substrate specificity of NAA10 is modulated by post-translational modifications, which motivated us to do this study.

Factor Inhibiting HIF (FIH), which belongs to the 2-oxoglutarate-dependent dioxygenase family, hydroxylates the Asn803 residue within the C-terminal transcriptional activation domain (CAD) of HIF-1α and this oxygen-dependent reaction occurs under normoxia. The Asn803 hydroxylation blocks the p300/CBP recruitment to CAD, thereby repressing the HIF-1-driven gene expression [17], [18]. Besides asparagine residue, the dioxygenase family is also able to hydroxylate β-carbon in aliphatic side-chain of lysine, aspartate, proline, arginine, and tryptophan residues [19]. Surprisingly, we here found that FIH hydroxylates NAA10 at Trp38 under normoxia. Moreover, the Trp38 hydroxylation renders NAA10 to widen the entrance gate of its catalytic pocket, leading to the entrance of a lysine residue into the pocket. It was also shown that W38-hydroxylated NAA10 can acetylate a lysine residue of HIF-1α but the unhydroxylated form cannot. The lysyl acetylation facilitates the E3 ubiquitin ligase pVHL to target HIF-1α for degradation under normoxia. This scenario may be a reasonable answer to the question about how monomeric NAA10 has the oxygen-dependent activity of lysine acetyltransferase.

## 2 Materials and methods

### 2.1 Cell culture

Human embryonic kidney (HEK293 and HEK293T), human breast cancer (MDA-MB-231) cell lines were obtained from American Type Culture Collection (ATCC; Manassas, VA). HEK293 and HEK293T cells were cultured in DMEM, MDA-MB-231 in RPMI1640, supplemented with 10% heat-inactivated fetal bovine serum (FBS). Cells were grown in a humidified atmosphere containing 5% CO_2_ at 37 °C. The ambient oxygen level was 20% for normoxia, 0.5% for hypoxia, or near 0.1% for anoxia.

### 2.2 Antibodies and reagents

Culture media, FBS, dimethyloxalylglycine (DMOG), and antibodies against FLAG-tag (F7425, 1:5000 dilution), and HA-tag (11867423, 1:10,000) were purchased from Sigma-Aldrich (St. Louis, MO). Antibodies against FIH (sc-271780, 1:5000 for Western blotting; sc-21219, 1:200 for Immunofluorescence), GST (sc-138, 1:10,000), β-tubulin (sc-9104, 1:5000), Lamin-B (sc-6216, 1:1000), VHL (sc-55506, 1:500) and HRP-conjugated goat anti-rat secondary antibody (sc-2006, 1:5000) were from Santa Cruz Biotechnology (Santa Cruz, CA). HRP-conjugated goat anti-rabbit (G21234, 1:5000), HRP-conjugated goat anti-mouse (G21040, 1:5000), Alexa Fluor 488 rabbit anti-goat (A11078, 1:1000) and Alexa Fluor 594 goat anti-rat (A11007, 1:1000) secondary antibodies were purchased from Invitrogen (Carlsbad, CA); anti-acetylated lysine antibody (9441, 1:4000) from Cell Signaling Technology (Danvers, MA); anti-His(6)-tag antibody (PM032, 1:1000) from MBL (Nagoya, Japan). Anti-HIF-1α (1:1000) and anti-NAA10 antibodies (1:1000 for Western blotting; 1:200 for Immunofluorescence) were generated in rabbits, as previously described [8], [20]. The antibody against N803-hydroxylated HIF-1α (1:1000) was a generous gift from Dr. Myung-Kyu Lee (Korea Research Institute of Bioscience & Biotechnology, Korea) [21].

### 2.3 Preparation of plasmids, siRNAs and transfection

The NAA10, FIH and HIF-1α plasmids were constructed, as previously described [8], [22]. Briefly, the cDNAs for human NAA10 (NM_003491) and human FIH (NM_017902.2) were cloned by RT-PCR using Pfu DNA polymerase and inserted into pcDNA, pcDNA-HA, pcDNA-FLAG, pcDNA-FLAG/SBP, pGEX, pET-28c-HIS plasmids by blunt-end ligation. The site-specific mutation of the NAA10 plasmid was performed using PCR-based mutagenesis (Stratagene; San Diego, CA). The plasmids for FLAG-tagged FIH fragments were constructed as previously described [22]. The plasmids for oxygen dependent degradation domain (ODDD; amino acids 401–603) and C-terminus (CT; amino acids 730-826) of human HIF-1α (NM_001530.3) were constructed by inserting the re-cloned cDNAs into pGEX vector [22]. For transient transfection of plasmids or siRNAs, cells at ~70% confluency were transfected using Lipofectamine 3000 reagent (for plasmid) or Lipofectamine RNAiMAX (for siRNA) reagent. The nucleotide sequences (5′ to 3′) of siRNAs used are; AGCUAUAACUGCGAACUGGGAUUCAU for silencing human FIH; CCACGAGCUUUCACAAUAAAUUCGC for silencing human NAA10; AUGAACGUGAAUUGCUCAATT for the non-targeting control.

### 2.4 Immunoblotting and immunoprecipitation

Proteins were separated on SDS-polyacrylamide gels and transferred to Immobilon-P membranes (Millipore; Bedford, MA). The membranes were blocked with 5% skim milk, incubated overnight at 4 °C with a primary antibody, and incubated with a horseradish peroxidase (HRP)-conjugated secondary antibody for 1 h, and visualized using the ECL-plus kit (Amersham Biosciences; Piscataway, NJ). To analyze protein interactions, cell lysates were incubated with anti-FIH, anti-NAA10, or IgG overnight at 4 °C, and the immune-complexes were pulled down with protein A/G beads (Santa Cruz Biotechnology). Otherwise, cell lysates were incubated with EZview Red anti-HA or anti-FLAG affinity gel (Sigma-Aldrich) at 4 °C for 4 h. The bound proteins were eluted in a denaturing SDS sample buffer or HA/FLAG-tag peptides, and loaded on SDS-PAGE. All Western blotting experiments were performed three or more times.

### 2.5 Fractionation of cytoplasmic and nuclear components

Cells were centrifuged at 1000 ×*g* for 5 min and the pellet was lysed in a buffer containing 10 mM Tris-HCl/pH 7.4, 10 mM KCl, 1 mM EDTA, 1.5 mM MgCl_2_, 0.2% NP-40, 0.5 mM dithiotheritol, 1 mM sodium orthovanadate, and 400 μM PMSF. The cell lysates were separated into pellet (for nuclear fraction) and supernatant (for cytosolic fraction) using centrifugation at 1000 ×*g* for 5 min. One packed volume of a nuclear extraction buffer (20 mM Tris–HCl/pH 7.4, 420 mM NaCl, 1 mM EDTA, 1.5 mM MgCl_2_, 20% glycerol, 0.5 mM dithiotheritol, 1 mM sodium orthovanadate, and 400 μM PMSF) was added to the pellet, and vortexed intermittently at low speed on ice for 30 min. The nuclear and cytosolic factions were spun at 20,000 ×**g** for 10 min and stored at −70 °C.

### 2.6 Immunofluorescence analysis

Cells were fixed with 3.7% formaldehyde for 10 min and permeabilized with 0.1% Triton X-100 for 30 min. Cells were incubated in PBS containing 0.05% Tween‐20 and 3% bovine serum albumin for 1 h, and further incubated overnight at 4 °C with a primary antibody. Cells were incubated with Alexa Fluor 488 or Alexa Fluor 594-conjugated secondary antibodies for 1 h. To stain nuclei, cells were incubated with DAPI (Sigma-Aldrich) for 30 min, and mounted in Faramount aqueous mounting medium (Dako; Glostrup, Denmark). Immunostained cells were observed under Carl Zeiss LSM510 META confocal microscope.

### 2.7 Preparation of recombinant proteins

Recombinant GST-FIH, GST-ODDD, GST-CT, and free GST proteins were expressed in *Escherichia coli* BL21 cells, pulled down using glutathione-affinity beads (GE Healthcare; Chicago, IL) at 4 °C for 1 h, and eluted with 10 mM reduced glutathione (Sigma-Aldrich). Recombinant His(6)-NAA10 WT and W38F proteins were expressed in BL21 cells, bound to Nickel-NTA affinity beads (Qiagen; Hilden, Germany) at 4 °C for 1 h, and eluted with 250 mM imidazole. The amounts and purities of extracted proteins were checked by SDS-PAGE and Coomassie Brilliant Blue R-250 staining.

### 2.8 *In vitro* binding assay

The mixtures of GST-FIH and His-NAA10 WT (or W38F) were incubated in a binding buffer (25 mM HEPES/pH 7.5, 150 mM KCl, 12.5 mM MgCl_2_, 0.5 mM dithiotheritol, 0.1% NP‐40, and 10% glycerol) at 4 °C for 1 h, and further incubated with glutathione or nickel affinity beads at 4 °C for 1 h. After the beads were washed with the binding buffer, bound proteins were eluted in the denaturing SDS sample buffer and subjected to Western blotting.

### 2.9 *In vitro* hydroxylation assay

*In vitro* hydroxylation assay was performed as previously described [23]. To prepare protein samples for LC-MS/MS analysis of NAA10, His-NAA10 and GST-FIH were incubated with hydroxylation cofactors (1 mM α-ketoglutarate, 1 mM ascorbate, and 100 mM FeSO_4_) in a hydroxylation buffer (40 mM Tris-HCl/pH7.5, 10 mM KCl, 3 mM MgCl_2_) at 37 °C for 1 h. His-NAA10 was electrophoresed, digested in gel, and analyzed by LC-MS/MS. For hydroxylation assay of HIF-1α CT, an acetyltransferase reaction was performed with GST-FIH and His-NAA10 at 37 °C for 1 h, followed by *in vitro* hydroxylase reaction with GST-HIF-1α CT and hydroxylation cofactors at 37 °C for 1 h. GST-HIF-1α CT was electrophoresed and immunoblotted with anti-N803(OH) HIF-1α antibody [21].

### 2.10 Mass spectrometric analysis

For LC-MS/MS analysis of intracellular NAA10 protein, HEK293 cells, which had been transfected with the FLAG/SBP-NAA10 plasmid, were treated with PBS or 500 μM DMOG for 24 h. FLAG/SBP-NAA10 was purified using FLAG-affinity beads and separated by SDS-PAGE. For LC-MS/MS analysis of *in vitro* hydroxylated NAA10, recombinant His-NAA10, which was reacted as described in the previous paragraph, was separated by SDS-PAGE. Proteins in gel slices were digested with trypsin and loaded to Easy n-LC (Thermo Fisher; San Jose, CA). Samples were separated on a C18 nanobore column (150 mm × 0.1 mm, 3 µm pore size; Agilent). The mobile phase A for LC separation was 0.1% formic acid, 3% acetonitrile in deionized water and the mobile phase B was 0.1% formic acid in acetonitrile. The chromatography gradient was designed for a linear increase from 5% B to 55% B in 40 min, 52% B to 75% B in 4 min, 95% B in 4 min, and 3% B in 6 min. The flow rate was maintained at 1500 nL/min. Mass spectra were analyzed using LTQ Orbitrap XL mass spectrometer (Thermo Fisher) equipped with a nano-electrospray source. Mass spectra were acquired using data-dependent acquisition with a full mass scan (350–1200 *m/z*) followed by 10 MS/MS scans. For MS1 full scans, the orbitrap resolution was 30,000 and the AGC was 2 × 10^5^. For MS/MS in the LTQ, the AGC was 1 × 10^4^. The hydroxylation was identified by the additional mass of 16 Da on digested peptides.

LC-MS/MS data files have been published in Mendeley Data and are available at https://data.mendeley.com/datasets/9b6xh36f3p/draft?a=f7ec8dac-5b5d-407b-b980-af2e78ab57f1

### 2.11 *In vitro* CO_2_ capture assay

The dioxygenase activity of FIH was measured using the CO_2_ capture assay, as previously described [23], [24]. Briefly, Recombinant GST-FIH and His-NAA10 WT (or W38F) were incubated with 1 mM ascorbate, 100 μM FeSO_4_, and 5 μl of [1–^14^C]-labeled 2-oxoglutarate with a specific activity of 58.7 mCi/mmole (Perkin-Elmer, Wellesley, MA) in a reaction buffer which contains 20 mM Tris-HCl/pH7.5, 0.5 mM dithiotheritol, 0.1% BSA, and 150 mM NaCl. A filter paper soaked in 30 mM Ca(OH)_2_ solution was immediately placed in the airtight screw-cap microcentrifuge tube. Each reaction was timed individually at 37 °C for 20 min and terminated by removal of filters. The hydroxylated level of NAA10 was measured by quantifying [^14^C]-labeled CO_2_ captured by the air dried filter paper using a liquid scintillation counter.

### 2.12 *In vitro* acetylation assay

GST-FIH (0.5 μg) was incubated with His-NAA10 (0.5 μg) in an acetylation buffer (50 mM Tris/pH 8.0, 0.1 mM EDTA, 1 mM dithiotheritol, 10 mM sodium butyrate, 400 μM acetyl CoA, and 5% glycerol) at 37 °C for 2 h. GST-FIH was pulled down using glutathione affinity beads, and subjected to Western blotting using anti-acetyl lysine antibody. For radio-isotopic assay, the mixture (20 μl) of 0.5 μg of His-NAA10 WT (or W38F) and 0.5 μg of GST-FIH were incubated with 1 mM α-ketoglutarate, 1 mM ascorbate, and 100 μM FeSO_4_ in the hydroxylation buffer at 37 °C for 30 min. After the reaction, GST-HIF-1α ODDD was added to the reaction mixture with the acetylation buffer as total volume of 60 μl, and further reacted with 200 μM [^14^C]-labeled acetyl CoA (60 mCi/mmole) and 200 μM unlabeled acetyl CoA at 37 °C for 2 h. The acetylated GST-HIF-1α ODDD was pulled down using glutathione affinity beads and its radioactivity was measured by a liquid scintillation counter.

### 2.13 Molecular dynamics simulation

All-atom atomistic MD models were used to simulate heterodimeric NAA10/NAA15 and monomeric NAA10 systems. The initial protein structures were obtained from the protein data bank (PDB ID code: 5nnp) [25] and NAA10-W38OH was prepared by hydroxylation of W38 residue at β-carbon position. Each of dimeric or monomer system with or without W38 hydroxylation was modeled to be solvated by water molecules with 100 mM NaCl in the simulation box with a periodic boundary condition. The CHARMM27 force field was used for natural amino acid residues while the force fields for W38OH was generated using CHARMM-GUI PDB Manipulator [26]. With this system constitution, NPT-ensemble MD simulations were carried out at 310 K and 1 bar using GROMACS 5.1.2 program [27]. The modified Berendsen thermostat [28] and Parrinello-Rahman barostat [29] were used for maintaining temperature (310 K) and the pressure (1 bar). A full system periodic electrostatics was employed by the particle-mesh Ewald method with a 1 Å grid spacing [30]. The cutoff and switching distances for van der Waals force were set to be 12 Å and 10 Å, respectively. The bonds involving hydrogen were constrained to be rigid by using the LINCS algorithm [31]. The MD system was equilibrated for 50 ns with a 2 fs time step and the structure analysis was done for simulating a further 50 ns run with recording every 2 ps.

### 2.14 Quantitative RT-PCR

Total RNAs were extracted using TRIZOL (Invitrogen; Carlsbad, CA), and reverse-transcribed in a reaction mixture containing M-MLV Reverse Transcriptase (Enzynomics, Daejeon, Korea), RNase inhibitor, dNTPs, and random primers at 42 °C for 60 min. Quantitative real-time PCR on 96-well optical plates was performed in the qPCR Mastermix (Enzynomics, Daejeon, Korea), and fluorescence emitting from dye-DNA complex was monitored in CFX Connect Real-Time Cycler (BIO-RAD, Hercules, CA). The mRNA values of targeted genes were calculated relative to GAPDH expression for each sample. All reactions were performed as triplicates. The nucleotide sequences (5′ to 3′) of PCR primers are; TTCTGAAAGTTTTCCTTCCA and TGTTGCAAGCTCAGAAGTAA for human *BNIP3*; CCTTTGAGGAAAATTGACAG and AACCCTCTAGGGAATACAGC for human *PDK1*; GGAAGAAAACAGTGCCTATG and GTGTAGTCAGAGACCCCTCA for human *CA9;* GAGTCAACGGATTTGGTCGT and TTGATTTTGGAGGGATCTCG for human *GAPDH*.

### 2.15 Statistical analysis

All data were analyzed using the Microsoft Excel 2013 or GraphPad Prism 5.0 software, and results were expressed as the means and standard deviation. The unpaired, two-sided Student's *t*-test was used to compare the results from *in vitro* assays, densitometry analyses of immunoblots, and quantitative RT-PCR. All statistical significances were considered when *P* values were less than 0.05.

## 3 Result

### 3.1 FIH interacts with NAA10

**Fig. 1:**
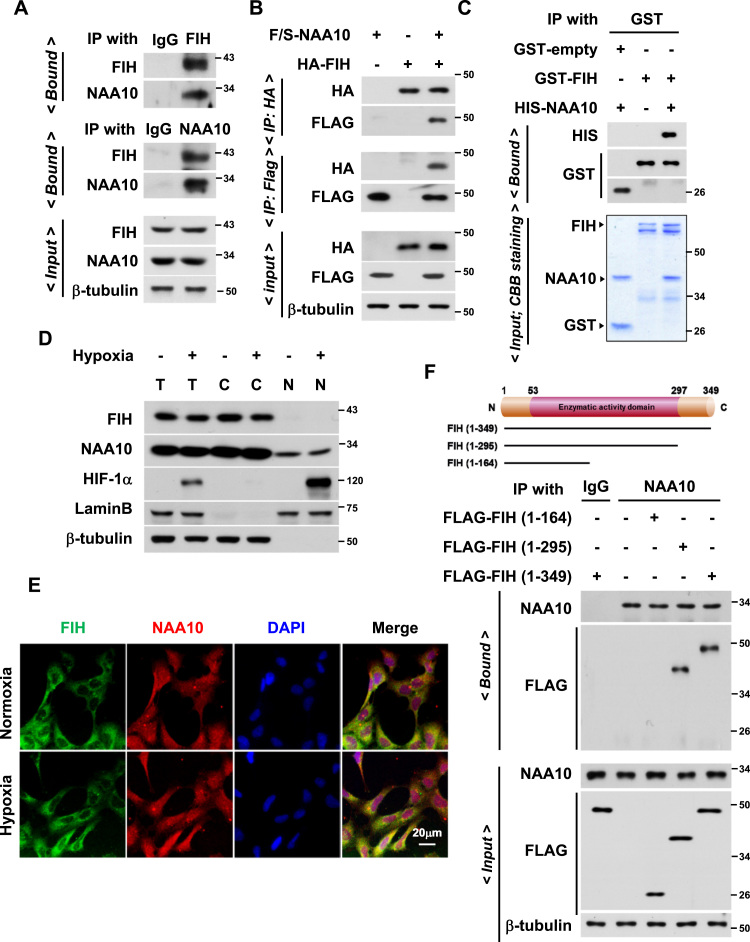
**FIH and NAA10 coexist in the cytoplasm and interact with each other.** (**A**) Proteins in HEK293 cell lysates were immunoprecipitated by anti-FIH or anti-NAA10 antibody and immunoblotted using the indicated antibodies. (**B**) HEK293 cells were transfected with HA-FIH (1 μg/ 60-mm dish) and/or FLAG/SBP-NAA10 (1 μg), and the lysates were subjected to immunoprecipitation using HA or FLAG affinity beads. (**C**) Recombinant proteins GST-FIH (0.5 μg) and His-NAA10 (0.5 μg) were co-incubated in 20 μl of a binding buffer for 1 h. The mixture was pulled down using glutathione affinity beads and immunoblotted (Top). Protein loading was verified by staining gels with Coomassie brilliant blue (Bottom) (**D**) HEK293 cells were exposed to normoxia (-) or hypoxia (+) for 24 h. Total lysate (T) was fractionated to cytosolic (C) and nuclear (N) components and the samples were immunoblotted. (**E**) HEK293 cells were cultured in normoxia or hypoxia for 24 h and subjected to immunofluorescence analysis using anti-FIH and anti-NAA10 antibodies. FIH and NAA10 were visualized with Alexa Fluor 488 (green) and Alexa Fluor 594 (red) conjugated secondary antibodies, respectively. Nuclei were stained with DAPI (blue). (**F**) Schematic diagram of FIH fragments (top). Each FIH fragment was expressed in HEK293 cells, and the cell lysates were subjected to immunoprecipitation and immunoblotting (bottom).

Recently, a report provided the mass spectrometry-based screening results about putative FIH substrates [32]. In this data base, interestingly, NAA10 was listed as a potential candidate for the FIH substrate. Given such information, we explored the physical interaction between FIH and NAA10. Either endogenous or ectopically expressed FIH and NAA10 proteins were co- precipitated with each other (Fig. 1A and B). In an *in vitro* binding analysis, recombinant His-NAA10 and GST-FIH proteins were pulled down together by glutathione affinity beads (Fig. 1C). Western blotting of subcellular fractions revealed that endogenous NAA10 and FIH are expressed in the cytoplasm regardless of oxygen tension (Fig. 1D). By analyzing confocal images, we found that FIH and NAA10 both are predominantly presented in the cytoplasm (Fig. 1E and Supplementary Fig. 1). To determine which domain of FIH interacts with NAA10, we performed co-immunoprecipitation analysis with three FIH fragments (amino acids 1-164, 1-295 and 1-349). The middle portion (165–295) of FIH, which includes dioxygenase catalytic domain, was identified to be essential for its binding to NAA10 (Fig. 1F). Taken together, FIH and NAA10 are likely to be associated probably in the cytoplasm.

### 3.2 NAA10 does not acetylate FIH post-translationally

Because NAA10 and FIH both have enzymatic functions, it was uncertain who plays an enzymatic role in the interaction between them. Given that the NAA10 acetylation of lysine residues has been reported [7], [8], [9], [10], [11], [12], we first checked the possibility that NAA10 acetylates FIH. We examined whether NAA10 overexpression or knock-down affects the level of acetylated FIH in HEK293, but found no difference in the acetylated FIH level (Supplementary Fig. S2A). In addition, a dominant-negative mutant NAA10 ΔCoA, which is defective in binding to acetyl CoA, did not change the acetylation status of FIH. Even though NAA10 was abundantly expressed, the FIH acetylation did not increase further (Supplementary Fig. S2B). In *in vitro* binding and acetylation assays, recombinant NAA10 interacted with recombinant FIH, but failed to acetylate FIH (Supplementary Fig. S2C). To test the possibility that the FIH activity to hydroxylate HIF-1α is regulated by NAA10, we carried out an *in vitro* hydroxylase reaction and measured the hydroxylated form of HIF-1α Asn803 using a specific antibody [21]. As expected, the FIH-catalyzed Asn803 hydroxylation was not affected by co-incubation with the NAA10 (Supplementary Fig. S2D). These results rule out the possibility that FIH is the substrate for NAA10.

### 3.3 FIH hydroxylates NAA10 at W38

**Fig. 2:**
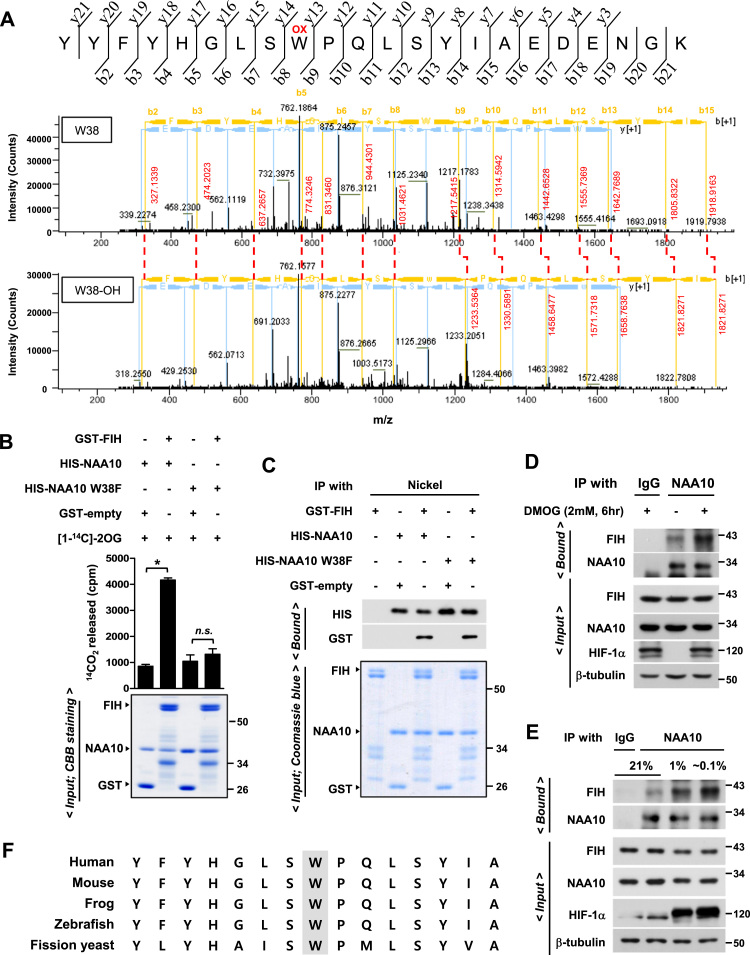
**FIH hydroxylates NAA10 at W38.** (**A**) HEK293 cells were transfected with the FLAG/SBP-NAA10 plasmid. After cells were treated with PBS (-DMOG) or DMOG for 24 h, FLAG/SBP-NAA10 was purified using FLAG affinity beads, electrophoresed, and digested in gel. Eluted proteins were subjected to LC-MS/MS analysis. The hydroxylation (OH) of W38 residue was identified based on the shift of peptide peaks. (**B**) *In vitro* hydroxylation assay. Recombinant His-NAA10 or W38F was reacted with recombinant GST-FIH in a hydroxylation assay buffer. The by-product, ^14^CO_2_ from the enzymatic reaction was captured and quantified using a scintillation counter. Each bar represents the mean + s.d. from 3 independent experiments (Top). *, P < 0.05; *n.s*., P > 0.05. Protein loading was verified by gel staining (Bottom). (**C**) *In vitro* binding assay. The recombinant proteins were put together in a test tube, and pulled down using nickel affinity beads. Western blotting (Top) and gel staining (Bottom) were carried out. (**D**) Co-immunoprecipitation of FIH and NAA10 was analyzed in HEK293 cells which had been treated with 2 mM DMOG for 6 h. (**E**) Co-immunoprecipitation of FIH and NAA10 was analyzed in HEK293 cells which had been incubated at the indicated oxygen level for 24 h. (**F**) Alignments of NAA10 amino acid sequences in human, mouse, frog, zebra fish, and fission yeast. The grey highlight indicates the tryptophan residues expected to be hydroxylated by FIH.

To examine if NAA10 is the substrate for FIH, we induced an *in vitro* hydroxylation using recombinant proteins, and performed LC-MS/MS analysis to identify the hydroxylated residues of NAA10. Based on + 16 Da mass shift of digested peptides, the tryptophan 38 (W38) residue alone was identified to be specifically hydroxylated by FIH (Supplementary Fig. S3A and 3B). To examine whether the W38 hydroxylation of NAA10 occurs in cells, we performed LC-MS/MS analyses in HEK293 cells that were treated with a vehicle (- DMOG) or an FIH inhibitor DMOG (+ DMOG). Under normoxic conditions, + 16 Da mass shift of the W38 residue in NAA10 was detected in 28 of total 106 peptides identified (~26% hydroxylation). In contrast, the W38 hydroxylation was detected to a lesser extent (~ 4%) in the presence of DMOG (Fig. 2A). To further check the involvement of FIH in the W38 hydroxylation, wild-type NAA10 and W38F-mutated proteins were applied to the *in vitro* CO_2_ capture assay, which is a biochemical method to measure the dioxygenase activity. Wild-type NAA10 generated CO_2_ as a result of decarboxylation of 2-oxoglutarate, but the W38F mutant did not (Fig. 2B). Given that the mutant still interacted with FIH (Fig. 2C), the W38 residue might be the hydroxylation site rather than the binding site for FIH. As previously reported [32], FIH tends to associate with its substrate for a longer time while the hydroxylase reaction is blocked. Likewise, the FIH-NAA10 interaction was reinforced under hydroxylation inhibitory conditions, such as DMOG treatment (Fig. 2D) or oxygen depletion (Fig. 2E), which also supports our notion that FIH hydroxylates NAA10. Homology analysis of the hydroxylation motif in NAA10 shows that W38 is highly conserved across species from yeast to human (Fig. 2F). The oxygen-dependent modulation of NAA10 activity seems to be evolutionally developed for biological adaptation to hypoxic milieu.

### 3.4 The W38 hydroxylation widens the substrate gate of NAA10

**Fig. 3:**
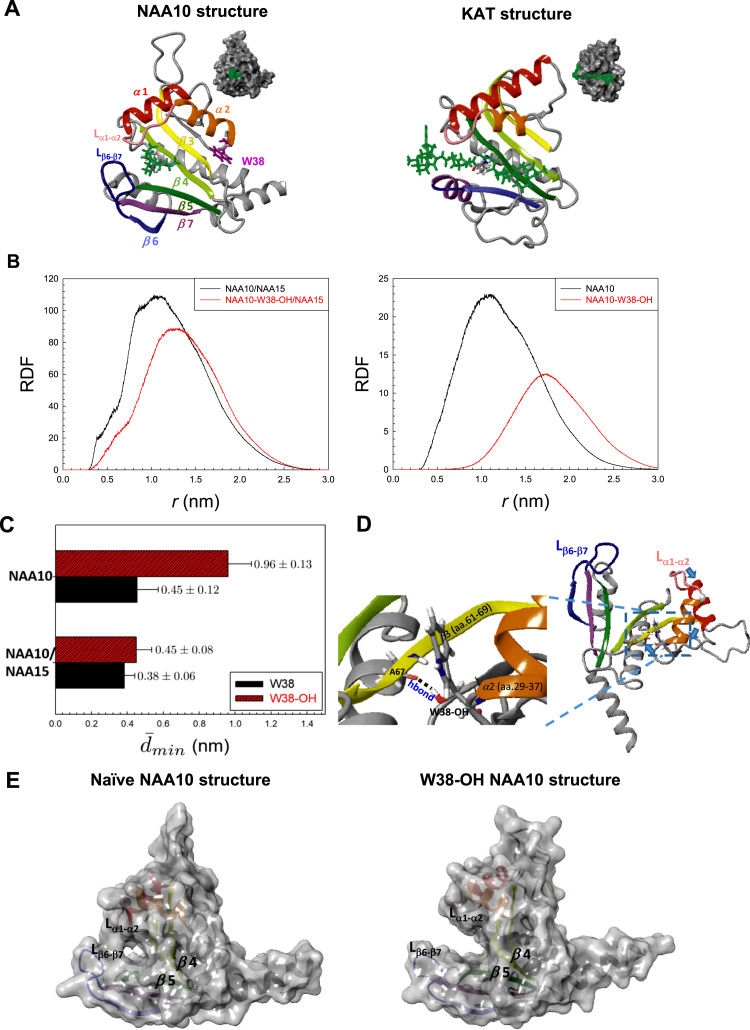
**W38-hydroxylation widens the substrate gate of NAA10.** (**A**) 3D structures of NAA10 (PDB code: 5nnp) with N-terminal serine residue (Left) and KAT (PDB code: 1qsn) with internal lysine residue (Right). The substrate peptides are presented as green sticks. The molecular surfaces in the upper right corners of 3D structures show that the substrate binding sites of NAA10 and KAT are shaped like cavity and groove, respectively. (**B**) The radial distribution functions (RDFs) of L_α1-α2_ with respect to L_β6-β7_ in the NAA10/NAA15 complex and in the W38-hydroxylated NAA10/NAA15 complex (Left) or those in the monomer of NAA10 and W38-hydroxylated NAA10 (Right). (**C**) The minimal interloop distance *d*_*min*_ is the shortest distance between L_β6-β7_ and L_α1-α2_. Each bar represents the mean + s.d. of the average of *d*_*min*_ in monomeric and dimeric NAA10. (**D**) Structure of W38-hydroxylated NAA10 monomer. The hydrogen bonding between a hydrogen in W38-OH and an oxygen in A67 carbonyl group bends L_α1-α2_ toward the arrow direction by pulling α2 helix. (**E**) Molecular surfaces of the monomeric NAA10 (Left) and W38-hydroxylated NAA10 (Right).

We next explored the functional consequences of FIH-mediated hydroxylation of NAA10. As shown in the amino acid sequences of NAA10 (Supplementary Fig. S4), the W38 residue is placed at an edge of the second α helix (α2, aa.29–37). In a view of crystal structure, NAA10 in association with NAA15 has a unique substrate-binding site like a cavity (Fig. 3A, Left), which is surrounded by α1–2 helices, β3–7 sheets, a loop between α1 and α2 (L_α1-α2_), and a loop between β6 and β7 (L_β6-β7_). The cavity-like shape is optimized for a specific accommodation of the N-terminal end of its substrates amino acids. In addition, L_β6-β7_ and L_α1-α2_ are placed at the entrance gate for target amino acids. Because the interloop distance is as narrow as 4 Å, NAA10 is not expected to catalyze lysine having a long side chain [5]. In comparison, the substrate-binding site of lysine acetyltransferase takes shape like a long groove with an open gate (Fig. 3A, Right), which allows an internal lysine reside to fit into the catalytic site. Since the W38 residue is present at the hinge of L_α1-α2_, we tested the possibility that the substrate-binding site gets changed in shape by the W38 hydroxylation. We employed an atomistic molecular dynamics (MD) model for simulating NAA10/NAA15 dimer and NAA10 monomer. The analyses of the dictionary of protein secondary structure (DSSP) (Supplementary Fig. S5A-D) and the root mean square deviation (RMSD) (Supplementary Fig. S6E and F) reveal that the W38 hydroxylation substantially alters protein conformation in monomer but less in dimer. This suggests that the structure of NAA10 gets more rigid in dimeric form with NAA15 than in monomer. In particular, the interloop distance between L_α1-α2_ and L_β6-β7_ becomes larger when W38 is hydroxylated and this trend is more pronounced in monomeric NAA10, as shown in radial distribution function (RDF) (Fig. 3B). The substrate gate opening by W38 hydroxylation was successfully simulated by the molecular dynamics program (supplementary movie S1 for W38-hydroxylated NAA10; movie S2 for non-hydroxylated NAA10). We also checked the minimal interloop distance *d*_*min*_, which is the distance between the closest pair of atoms where one belongs to L_β6-β7_ and the other belongs to L_α1-α2_ (Supplementary Fig. S5G and H). The average of *d*_*min*_ $\left({\bar{d}}_{\min}\right)$ in the monomeric NAA10 with W38-OH is 9.6 ± 1.3 Å, which is more than two times larger than that in the monomeric NAA10 with native W38 (4.5 ± 1.2 Å) (Fig. 3C). Upon closely inspecting the structure of W38-hydroxylated NAA10 (Fig. 3D), the hydrogen bonding between W38-OH and carbonyl oxygen of A67 (belonging to β3) bends L_α1-α2_ backward, which eventually widens the substrate gate enough to accommodate a lysine residue. In addition, the W38 hydroxylation transforms the substrate-binding site from cavity shape to long groove one (Fig. 3E). It is expected that a cavity-like gate may be suitable to put a protein end in it and a glove-like one to bite an internal strand of protein. Therefore, W38-hydroxylated NAA10 seems to have a structure suitable for acetylation of an internal lysine residue.

Supplementary material related to this article can be found online at doi:10.1016/j.redox.2018.09.002.

The following is the Supplementary material related to this article Movie 1, Movie 2.

Movie 1 **Molecular dynamics (MD) simulation of W38-hydroxylated NAA10.** The substrate gate opening by W38 hydroxylation was simulated using GROMACS program. Acetyl CoA was presented as skeletal molecular model and catalytic pocket of NAA10 as ribbon diagram. L_α1-α2_ and L_β6-β7_ were presented as green and blue loops, respectively. α2 helix was presented as yellow ribbon and β3 sheet was presented as orange ribbon. The interloop distance between L_α1-α2_ and L_β6-β7_ becomes larger when W38 is hydroxylated. The movie is related to main Fig. 3B.

Movie 2 **Molecular dynamics simulation of non-hydroxylated NAA10.** The substrate gate of non-hydroxylated NAA10 was simulated using GROMACS program. Acetyl CoA was presented as skeletal molecular model and catalytic pocket of NAA10 as ribbon diagram. L_α1-α2_ and L_β6-β7_ were presented as green and blue loops, respectively. α2 helix was presented as yellow ribbon and β3 sheet was presented as orange ribbon. The interloop distance between L_α1-α2_ and L_β6-β7_ of non-hydroxylated NAA10 is narrow than of W38-hydroxylated NAA10. The movie is related to main Fig. 3B.

### 3.5 The W38 hydroxylation transforms NAA10 to acetylate lysine

**Fig. 4:**
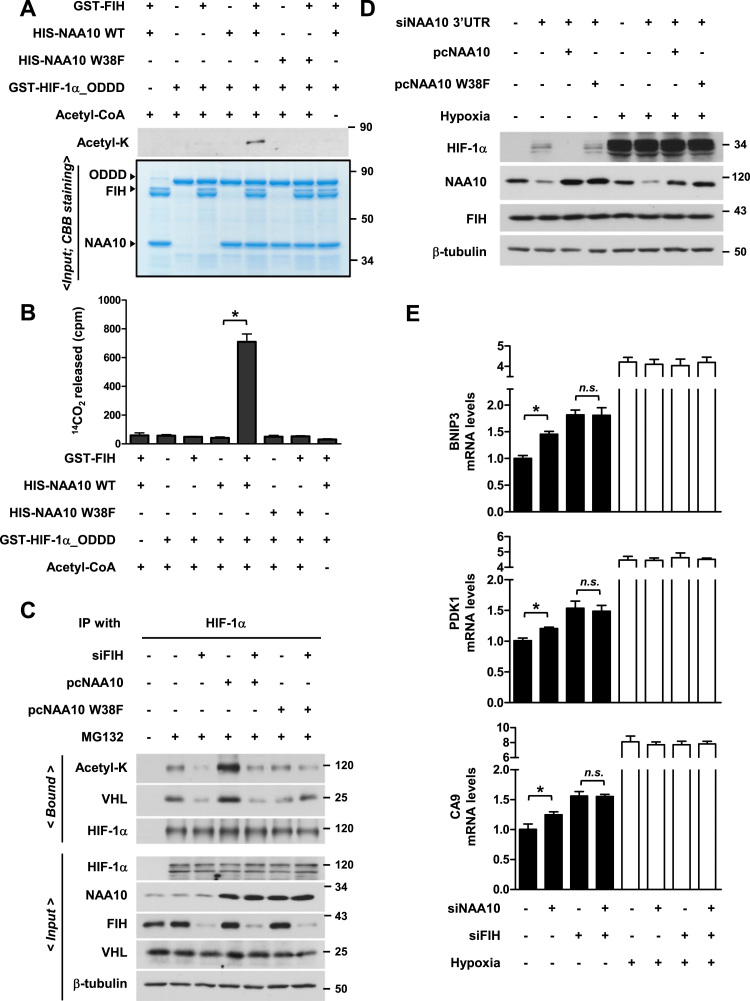
**W38-hydroxylation enables NAA10 to acquire a lysyl acetyltransferase activity for HIF-1α. (A**) *In vitro* acetylation assay. Firstly, recombinant GST-FIH and His-NAA10 (or W38F) were reacted in an *in vitro* hydroxylation buffer. Then, GST-HIF-1α ODDD was added as indicated, and further reacted with or without acetyl CoA. Acetylated HIF-1α was detected with anti-acetyl lysine antibody (Top). Protein loading was confirmed by staining gels with Coomassie brilliant blue (Bottom) (**B**) *In vitro* acetylation assay using radio isotope. Recombinant proteins were mixed as described in Fig. 4*A*, and reacted with 200 μM ^14^C-labeled acetyl CoA and 200 μM cold one. Acetylated HIF-1α was analyzed by scintillation counter. (**C**) HEK293 cells were co-transfected with the NAA10 (or W38F) plasmid and FIH siRNA (or control siRNA). After treated with 10 μM MG132 for 6 h, cells were subjected to immunoprecipitation with anti-HIF-1α antibody and immunoblotting with the indicated antibodies. (**D**) HEK293 cells were transfected with a siRNA targeting 3′UTR of NAA10 and/or the NAA10 (or W38F) plasmid. After incubated under normoxia or hypoxia (0.5% O_2_) for 6 h, cells were subjected to immunoblotting. (**E**) The mRNA levels of *BNIP3, PDK1,* and *CA9* were quantified by RT-qPCR (n = 3). HEK293 cells which had been transfected with NAA10 and/or FIH siRNAs were incubated under hypoxia (0.5% O_2_) for 24 h.

The FIH inactivation of HIF-1α through N803 hydroxylation has been well established [17], [18]. It was also proposed that NAA10 destabilizes HIF-1α through K532 acetylation under normoxia but does not upon NAA10 downregulation under hypoxia [7]. However, this scenario is challenged by other groups arguing that NAA10 neither decreases during hypoxia nor acetylates the lysine residue of HIF-1α [5], [13], [14], [15]. In our experimental settings, the knock-down of NAA10, not of NAA15, upregulated HIF-1α under normoxia but the expression of NAA10 was not reduced under hypoxia in HEK293 (Supplementary Fig. S6A). Compared with control cells, FIH knock-down cells expressed HIF-1α at a higher level even in mild hypoxia (> 1% oxygen) (Supplementary Fig. S6B and S6C). Unless NAA10 is hydroxylated, the oxygen-dependent destruction of HIF-1α might be blunted. Therefore, we hypothesized that FIH promotes the NAA10-mediated HIF-1α acetylation by hydroxylating NAA10 at W38 and by doing so plays an ancillary role in the PHD-initiated HIF-1α degradation under normoxia. To test this hypothesis, the acetyltransferase activity of NAA10 for HIF-1α oxygen dependent degradation domain (ODDD; a.a. 401–603) containing K532 was analyzed by Western blotting with anti-acetyl lysine antibody. As expected, we observed that the ODDD was acetylated *in vitro* only by FIH-hydroxylated NAA10, but not by the NAA10 W38F mutant (Fig. 4A). To recheck the NAA10 activity, we performed another acetylation assay using C^14^-labeled acetyl CoA. After NAA10 was pre-incubated with FIH, it catalyzed the acetylation of ODDD, whereas the W38F mutant did not even after FIH reaction (Fig. 4B). These findings strongly indicate that the FIH-mediated W38-hydroxylation of NAA10 is an essential prerequisite for the lysyl acetylation of HIF-1α ODDD. Since the HIF-1α K532-acetylation by NAA10 is known to enhance the pVHL binding to HIF-1α [7], we examined whether FIH promotes this interaction through NAA10 W38-hydroxylation in HEK293 and a human breast cancer cell line MDA-MB-231, which was used as a representative cancer cell. To stabilize HIF-1α under normoxia, cells were treated with a proteasome inhibitor MG132 and subjected to co-immunoprecipitation with anti-HIF-1α antibody. The overexpression of NAA10, but not of W38F, increased the level of lysyl-acetylated HIF-1α. However, FIH knock-down attenuated such an effect of NAA10. Accompanying the acetylation, NAA10 facilitated the interaction between pVHL and HIF-1α, which was demoted by FIH knock-down (Fig. 4C and Supplementary Fig. S6D). Given that the hydroxylated NAA10 promotes the pVHL targeting to HIF-1α, NAA10 might participate in HIF-1α degradation under normoxia. Surprisingly, the NAA10 knock-down by a 3′UTR-targeting siRNA blocked the normoxic degradation of HIF-1α. The HIF-1α stabilization was reversed by restoration of NAA10, but not by expression of W38F, in HEK293 (Fig. 4D) and MDA-MB-231 (Supplementary Fig. S6E) cells. However, this effect of NAA10 was not observed under hypoxia in both cell lines. Next, we examined whether HIF-downstream genes are regulated through the FIH/NAA10-mediated destabilization of HIF-1α. Under normoxia, the transcripts of *BNIP3, PDK1* and *CA9* genes were upregulated by NAA10 knockdown (Fig. 4E). When FIH was silenced, the target gene expressions were enhanced due to the blockade of HIF-1α N803 hydroxylation. However, they were no more regulated by NAA10 knockdown because NAA10 was not capable of acetylating HIF-1α without FIH. Under hypoxia, the target genes were highly expressed regardless of NAA10 or FIH expression (Fig. 4E). These findings suggest that FIH is indirectly involved in the oxygen-dependent degradation of HIF-1α by hydroxylating NAA10 to get the lysine-acetyltransferase activity. The FIH-NAA10 axis seems to ensure the repression of hypoxia-induced genes under normoxic conditions.

## 4 Discussion

**Fig. 5:**
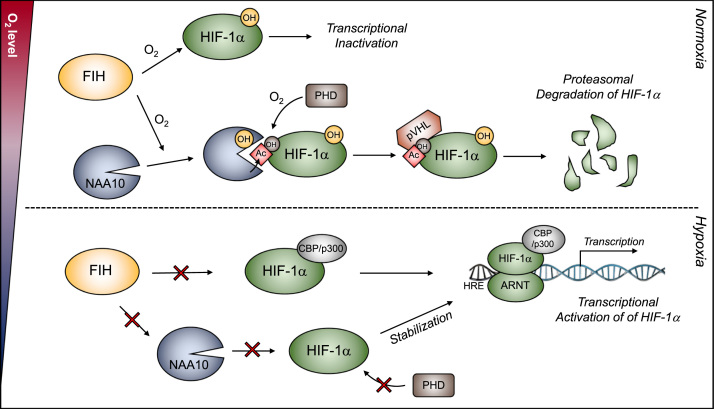
**Summary of PHD and FIH-mediated, oxygen-dependent regulation of HIF-1α.** Under normoxia, FIH hydroxylates the W38 residue of NAA10 by utilizing O_2_, which widens the substrate gate of NAA10 to acetylate the K532 residue of HIF-1α. The Lys-acetylation in association with PHD-mediated prolyl hydroxylation recruits the pVHL/E3 ubiquitin ligase complex, leading to HIF-1α degradation through the ubiquitin-proteasome system. FIH also hydroxylates the N803 residue of HIF-1α, which inhibits the transcriptional activity HIF-1α by blocking the recruitment of CBP/P300 coactivators. Under hypoxia, the FIH actions for NAA10 W38-hydroxylation and HIF-1α N803-hydroxylation are limited and consequently HIF-1α is stabilized and activated, thereby promoting the expression of hypoxia response genes.

Post-translational modification (PTM) of proteins plays a significant role in regulating cellular processes. Several reports showed that PTM of proteins are largely regulated in response to chronic sustained and intermittent forms of hypoxic stress [33], [34]. In case of HIF-1α, proline- and asparagine-hydroxylation have been intensively investigated as oxygen sensing mechanisms [17], [35]. Under normoxia, prolyl hydroxylase domain-containing proteins (PHD1–3) hydroxylate HIF-1α at P402 and P564, leading to pVHL-mediated poly-ubiquitination and subsequent degradation of HIF-1α. In addition, the transcriptional activity of HIF-1α is oxygen-dependently controlled through the FIH-mediated asparagine-hydroxylation. To date, PHD and FIH have been known to exclusively control HIF-1α in terms of stability and functionality, respectively. However, this paradigm about oxygen sensing mechanisms might be revised because FIH can control the stability of HIF-1α protein as well by modulating the substrate specificity of NAA10. In Fig. 5, we summarize the hypothetical mechanism about the PHD and FIH-mediated, oxygen-dependent regulation of HIF-1α.

In respect of the oxygen-dependent regulation of HIF-1α stability by NAA10, two questions have not been answered for two last decades. One is the acetyltransferase activity of NAA10 for HIF-1α. Based on biochemical data showing that NAA10 failed to acetylate HIF-1α *in vitro*, several research groups refuted this hypothesis [5], [13], [14], [15]. However, as they used the recombinant protein of non-hydroxylated NAA10 for the *in vitro* assay, it is obvious that NAA10 did not acetylate HIF-1α. After NAA10 is hydroxylated by FIH, it gets a new ability to acetylate HIF-1α even *in vitro*, which may be the answer to this question. The other is how the HIF-1α acetylation is regulated in an oxygen-dependent fashion. As a mechanism underlying this regulation, the downregulation of NAA10 under hypoxia was suggested [7]. However, this hypothesis was also challenged because the NAA10 level was not substantially reduced in hypoxic cells [14]. We here suggest that the acetyltransferase activity of NAA10 is reduced under hypoxia due to the limitation of FIH-mediated NAA10 hydroxylation. This may be a reasonable answer to the second question.

We here propose that the FIH-mediated W38 hydroxylation widely opens the entrance gate to admit the lysine into the pocket. This molecular dynamics model was also verified by biochemistry- and cell biology-based experiments for HIF-1α. In view of molecular dynamics, other substrates, which were previously known to be lysyl-acetylated by NAA10 [7], [8], [9], [10], [11], [12], are expected to be acetylated in an oxygen-dependent manner by NAA10. Considering that the NAA10-acetylated substrates regulate diverse cell signaling pathways, the FIH-NAA10 axis may be involved in many cellular responses to changing oxygen levels. This issue remains to be investigated.

FIH was originally identified to hydroxylate HIF-1α at N803 [17], [18]. Later, it has been reported that FIH also catalyzes various Ankyrin Repeat Domain (ARD)-containing proteins, such as Notch1–3, IkB-α, ASPP2, TRPV3, OTUB1, and G9a/GLP [23], [36], [37], [38], [39], [40]. A recent report searched for potential substrates of FIH and PHDs using quantitative interaction proteomics [32]. Of 192 putative FIH substrates in total, 112 proteins have neither ARD nor known consensus sequences for FIH binding. Indeed, we found that FIH hydroxylates NAA10 at a tryptophan residue rather than an asparagine residue. FIH seems to be a dioxygenase with a wider spectrum of substrates than we thought. Indeed, the 2-OG-dependent dioxygenase family, which is one of the biggest enzymes groups, catalyzes a variety of oxidative reactions [41]. Given that only a few residues (-8, -1, +10 residue to the hydroxylated position) are conserved at a part of FIH-hydroxylated substrates, FIH is regarded to target a wide spectrum of substrates including ARD-containing proteins [42]. To date, however, the FIH-hydroxylated residues have been known to be asparagine, aspartate [43], and histidine residues [44]. Therefore, it was surprising that FIH hydroxylates a tryptophan residue of NAA10. This result further expands the scope of FIH-catalyzed hydroxylation.

In conclusion, our results suggest that the FIH-mediated hydroxylation of NAA10 at W38 is a critical step in the oxygen-dependent regulation of HIF-1α stability through lysyl acetylation. This is a novel oxygen-sensing process that modulates the substrate specificity of enzyme depending on ambient oxygen tension.
