# Supplementary material for: FIH permits NAA10 to catalyze the oxygen-dependent lysyl-acetylation of HIF-1α
Source: Redox Biol. 2018 Sep 7;19:364–74. doi: 10.1016/j.redox.2018.09.002 (PMC6142190; doi:10.1016/j.redox.2018.09.002)
Supplement: Supplementary file 3 — Supplementary material [file mmc1.pdf]

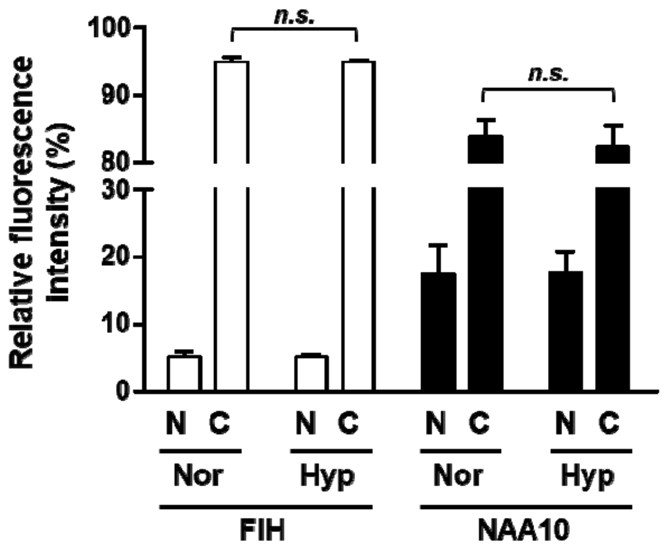

Figure S1. Subcellular localization of FIH and NAA10 is not changed by hypoxia.
Confocal images in Fig. 1E are analyzed with Image J plugin. Immunofluorescence intensities of nucleus and total cells were quantitated in three cells per image. Intensities of cytoplasm were calculated by subtraction of nucleus intensity from total cell intensity. (N; nucleus, C; cytoplasm, Nor; 21% oxygen, Hyp; 0.5% oxygen)

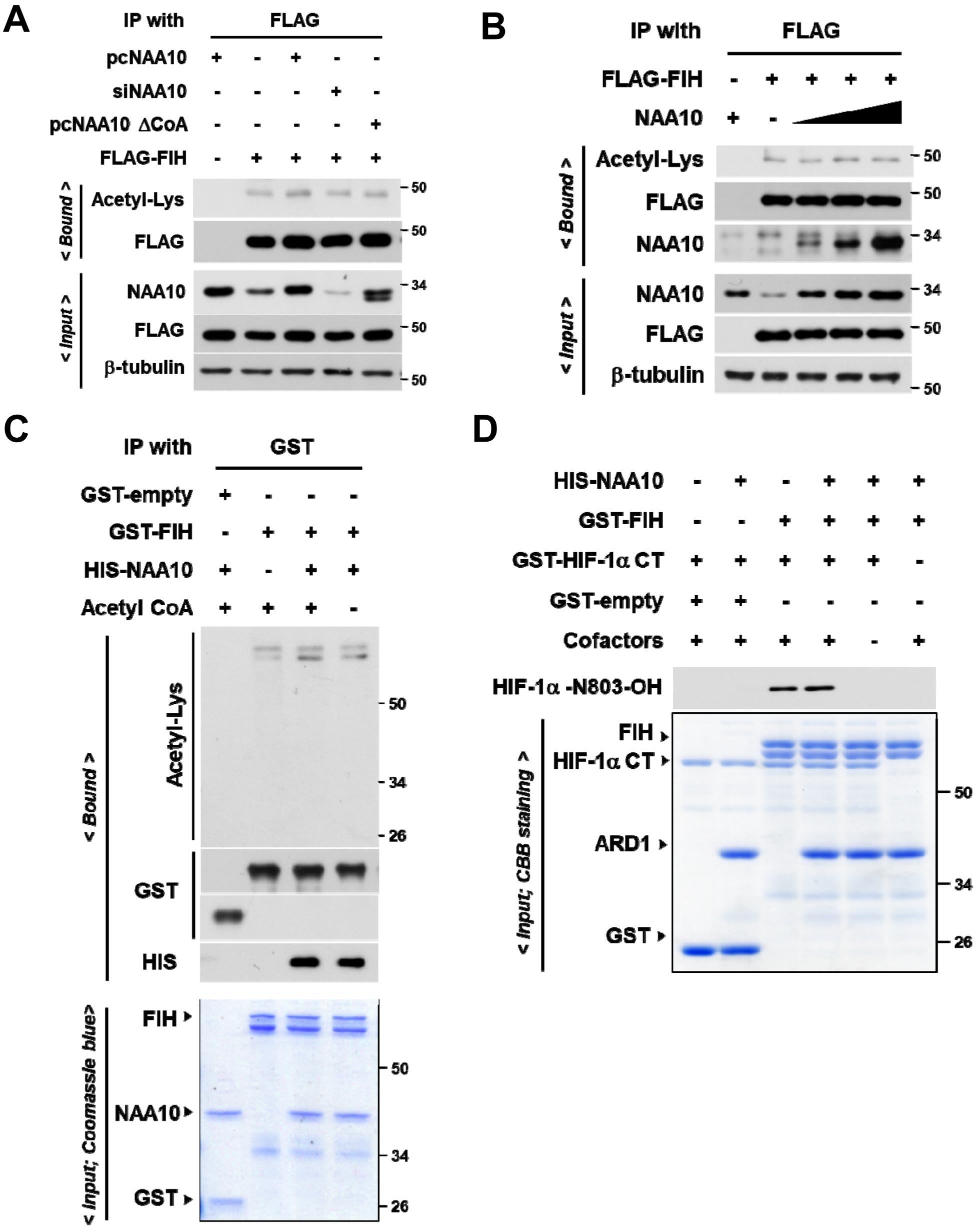

Figure S2. FIH is not a substrate of NAA10.

A. HEK293 cells were transfected as indicated. FLAG-FIH was immunoprecipitated using FLAG affinity beads and immunoblotted with the indicated antibodies.

B. HEK293 cells were co-transfected with FLAG-FIH and NAA10. FLAG-FIH was immunoprecipitated and immunoblotted.

C. Recombinant peptides GST-FIH and HIS-NAA10 were reacted with acetyl-CoA at 37°C for 2 hr, and GST-FIH was pulled down with GSH affinity beads. Acetylated FIH was analyzed using anti-acetyl Lys antibody (Top). Peptide input in reaction mixture was verified by Coomassie blue staining (Bottom).

D. GST-FIH, HIS-NAA10, GST-HIF-1α C-terminus, and free GST peptides were mixed as indicated, and reacted with cofactors at 37°C for 1 hr. N803-hydroxylation of HIF-1α C-terminus was analyzed using a monoclonal antibody against hydroxylated N803 residue (Top). Peptides on PAGE were stained with Coomassie brilliant blue (Bottom).

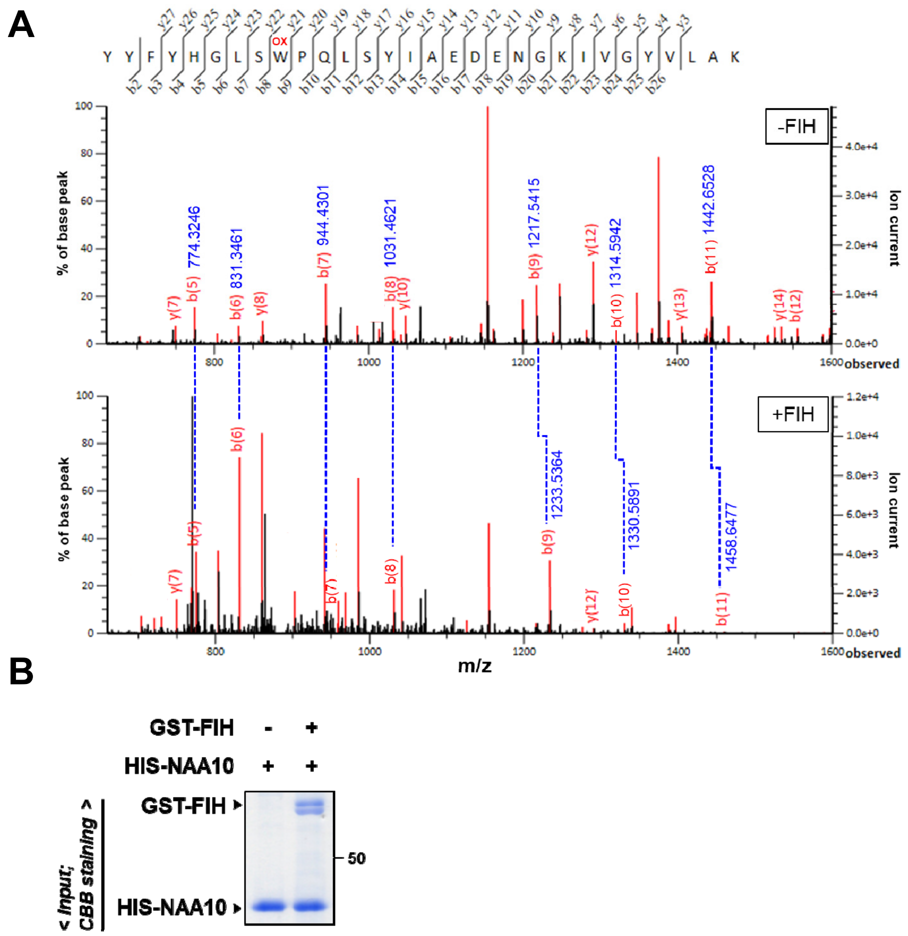

Figure S3. LC-MS/MS spectra for NAA10.

A. Recombinant peptides HIS-NAA10 was reacted with or without GST-FIH in a hydroxylation assay buffer at 37°C for 1 hr. HIS-NAA10 was electrophoresed, digested in gel, and subjected to LC-MS/MS.

B. Peptide input of *in vitro* hydroxylation assay for LC-MS/MS.

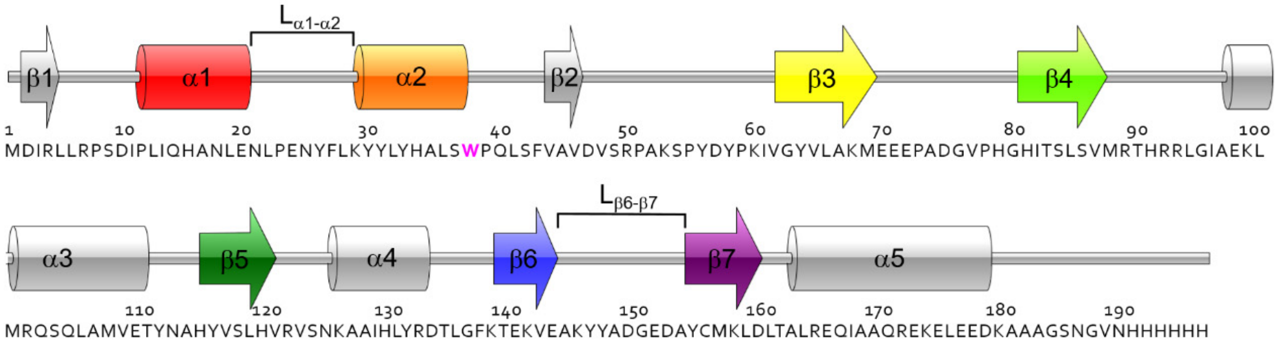

Figure S4. The primary and secondary structures of NAA10.
Secondary structural elements, from the crystal structure of NAA10/NAA15 complex (PDB code: 5nnp), are indicated above the sequences where α-helices and β-sheets are represented by cylinders and arrows, respectively.

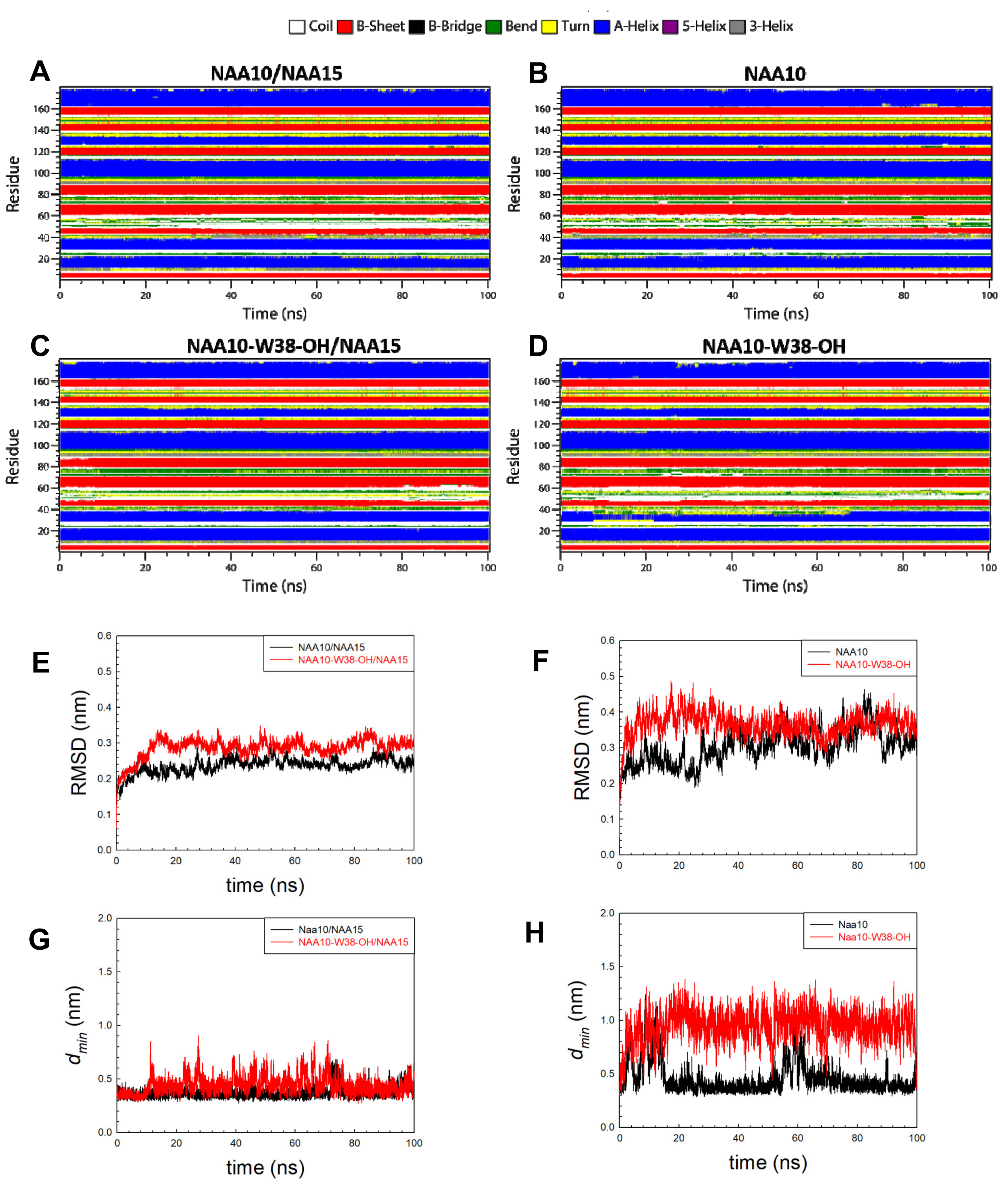

Figure S5. Molecular structural analysis of NAA10 and NAA10/NAA15 upon W38-hydroxylation.

A-D. The dictionary of protein secondary structure (DSSP) analysis for (*A*) NAA10 in the dimer with NAA15, (*B*) NAA10 in the monomer, (*C*) NAA10-W38OH in the dimer, and (*D*) NAA10-W38OH in the monomer.

E-H. The root mean square deviation (RMSD) from crystallographic data for NAA10 with and without W38-hydroxylation (*E*) in the dimeric, and (*F*) in the monomeric systems, and the minimal interloop distance d_{min} between $L_{\beta6\text{-}\beta7}$ and $L_{\alpha1\text{-}\alpha2}$ as a function of the MD simulation time for NAA10 with and without W38-hydroxylation (*G*) in the dimeric, and (*H*) in the monomeric systems.

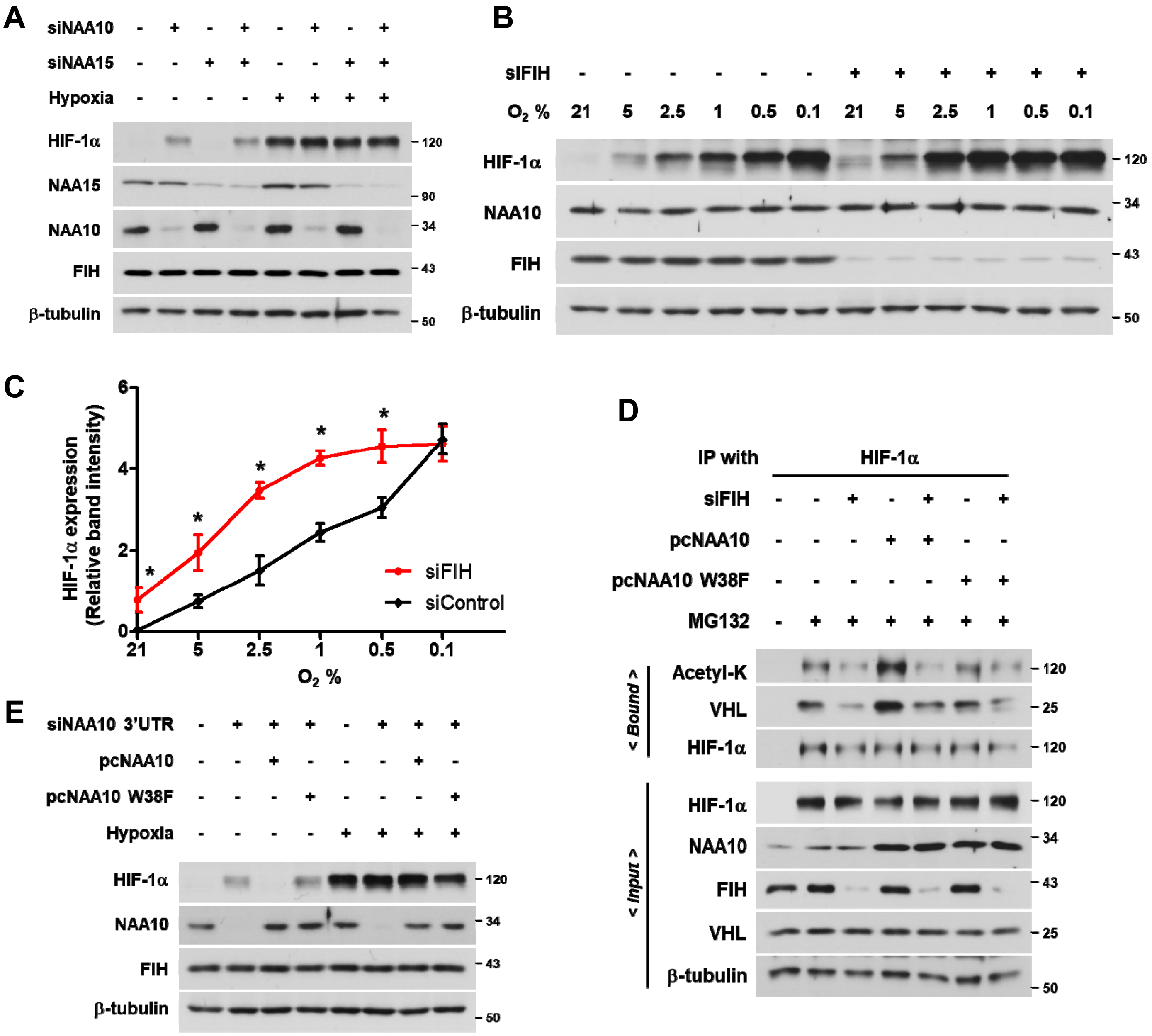

Figure S6. HIF-1α is partially stabilized by knocking-down either NAA10 or FIH.

A. MDA-MB-231 cells, in which NAA10 or NAA15 was knocked down, were incubated under normoxia or hypoxia (0.5% oxygen) for 4 hr, and subjected to immunoblotting.

B. MDA-MB-231 cells, which had been transfected with control or FIH siRNA, were incubated at the indicated oxygen tension for 4 hr, and subjected to immunoblotting.

C. The intensities of HIF-1α and β-tubulin immunoblots in MDA-MB-231 cells were measured using the ImageJ software. HIF-1α intensity in each condition was divided by β-tubulin intensity in the corresponding sample to normalize protein expression. Each symbol represents the mean ± s.d. ($n = 3$) and * denotes $p < 0.05$ versus the siControl group at the same oxygen level.

D. MDA-MB-231 cells were co-transfected with the NNAA10 (or W38F) plasmid and FIH siRNA (or control siRNA). After treated with 10 μM MG132 for 6 hr, cells were subjected to immunoprecipitation with anti-HIF-1α antibody and immunoblotting with the indicated antibodies.

E. MDA-MB-231 cells were transfected with a siRNA targeting 3'UTR of NAA10 and/or the NAA10 (or W38F) plasmid. After incubated under normoxia or hypoxia (0.5% O2) for 4 hr, cells were subjected to immunoblotting.
